# Radiofrequency Ablation a Safe and Effective Treatment for Pediatric Benign Nodular Thyroid Goiter

**DOI:** 10.3389/fped.2021.753343

**Published:** 2021-11-26

**Authors:** An-Ni Lin, Wei-Che Lin, Kai-Lun Cheng, Sheng-Dean Luo, Pi-Ling Chiang, Wei-Chih Chen, Yueh-Sheng Chen, Cheng-Kang Wang, Na-Ning Kan, Yan-Ye Su

**Affiliations:** ^1^Department of Diagnostic Radiology, Kaohsiung Chang Gung Memorial Hospital, Chang Gung University College of Medicine, Kaohsiung, Taiwan; ^2^Department of Medical Imaging, Chung Shan Medical University Hospital, Taichung, Taiwan; ^3^School of Medical Imaging and Radiological Sciences, Chung Shan Medical University, Taichung, Taiwan; ^4^Department of Otolaryngology, Kaohsiung Chang Gung Memorial Hospital, Chang Gung University College of Medicine, Kaohsiung, Taiwan

**Keywords:** radiofrequency ablation, thyroid gland, children, pediatric, goiter, ultrasound

## Abstract

**Purpose:** To evaluate the effectiveness of radiofrequency ablation (RFA) for benign thyroid nodules in pediatric patients.

**Materials and Methods:** Twelve pediatric patients (11 female, 1 male; mean age 15.54 ± 2.8 years, range 10–19 years) with benign thyroid nodules (mean longest diameter 4.1 ± 1.4 cm, range 1.5–5.9 cm) treated by RFA from 2017 to 2020 were evaluated. The inclusion criteria for RFA therapy were (i) age < 20 years; (ii) benign cytological confirmation by 2 separate ultrasound guided fine-needle aspiration cytology (FNAC) or core needle biopsies; (iii) pressure symptoms or cosmetic problems caused by thyroid nodules; (iv) absence of any sonographic suspicious feature; and (v) follow-up for >6 months. Under local anesthesia, RFA was performed with the use of an RF generator and an 18-gauge internally cooled electrode. Volume changes in nodules on follow-up ultrasonography (US), changes in symptomatic and cosmetic scores, and complications arising during or after RFA were evaluated.

**Results:** Mean follow-up period was 24.9 ± 13.9 months (range 6–43 months). At the last follow-up visits, volume of the nodule had decreased significantly (15.34 ± 11.52 mL vs. 4.07 ± 4.99 mL; *P* < 0.05), whereas volume reduction rate was 74.31% ± 19.59%. Both cosmetic and compressive symptoms were also significantly improved (2.91 ± 0.79 vs. 0.92 ± 0.67 and 1.5 ± 1.93 vs. 0.17 ± 0.39; *P* < 0.05). The mean number of ablation sessions was 1.4 ± 0.6 (range 1–3 sessions), and one of the patients suffered from transient vocal cord palsy which was spontaneously resolved 53 days later.

**Conclusions:** RFA is a safe and effective treatment for benign thyroid nodules in pediatric patients, and can thus serve as an alternative treatment for thyroidectomy.

## Introduction

Thyroid nodules are commonly diagnosed in adults, although they affect only 1–2% of the pediatric population based on clinical examinations (1). The pathophysiology of non-neoplastic thyroid nodule development in adolescents is associated with an increased production of thyroid hormone coinciding with pubertal development. In addition, relative iodine deficiency or persisted overstimulation of the thyroid gland with high-normal thyroid stimulating hormone (TSH) may initiate development of thyroid nodules with benign characteristics. Further, a thyroid growth spurt coinciding with menarche in females may contribute to a higher incidence of goiter during mid- to late-puberty stages (2, 3).

According to management guidelines for children with thyroid nodules and differentiated thyroid cancer, lobectomy may be performed in patients with compressive symptoms and cosmetic concerns or according to patient/parent preference and should be considered in all apparently benign solid thyroid nodules >4 cm (4). The primary risks of thyroidectomy include recurrent laryngeal or superior laryngeal nerve injury and hypoparathyroidism, hypocalcemia, bleeding, and postoperative infection (5, 6). Of note, pediatric patients who have undergone thyroidectomy have higher complication rates than adult patients (9.1 vs. 6.3%; *P* < 0.01) (7). Preservation of thyroid function is currently a prevailing concept in the treatment of benign thyroid disease. Patients receiving total thyroidectomy early in life require lifelong hormone substitution; however, it has been reported that over 40% patients are not able to completely adhere to postoperative medications (8). Children with primary hypothyroidism may present with poor linear growth, delayed puberty or precocious puberty (9). In patients followed for more than 1 year, 40% reported psychological or behavioral problems and concomitant abnormal thyroid function, suggesting clinically evident hypothyroidism (8). Furthermore, as traditional total thyroidectomy causes visible scarring at the anterior neck, patients could experience dermatologic symptoms such as pruritus, skin tightness, and pain, which negatively impact quality of life (10).

Pediatric patients with benign non-functioning thyroid nodules may encounter a challenging decision-making process due to the complexity of disease information, and the uncertainty of the risk-benefit analysis when they are young. The emerging importance of shared decision making (SDM) helps patients by clarifying treatment options to reduce the uncertainty of management decisions and improve health-related quality of life (11, 12). Several alternatives to surgery have been evaluated in adults, such as radiofrequency ablation (RFA), microwave ablation, and laser ablation, which offer treatment options for patients at high anesthetic risk or not suitable candidates for surgery (13). Current guidelines suggest that RFA could be considered for the treatment of benign symptomatic thyroid nodules in adults, with a high-volume reduction rate (range: 64.9–93.9%), improved cosmetic and symptoms scores, and low overall complication rates (2.11%) (13, 14). However, data regarding thyroid RFA in pediatric patients remains limited.

The purpose of our study was to evaluate the safety and effectiveness of RFA for benign thyroid nodules in pediatric patients. We also discuss the factors that might alter the pre-procedural planning and the variables that may affect treatment results.

## Materials and Methods

### Patients

Our study population consisted of 12 pediatric patients who underwent RFA for benign non-functioning thyroid nodules in Kaohsiung Chang Gung memorial hospital (10 patients) and Chung Shan Medical University Hospital (2 patients) from August 2017 to September 2020. All 12 patients fulfilled the following criteria: (i) age <20 years; (ii) at least 2 separate ultrasound guided fine-needle aspiration cytology (FNAC) or core needle biopsies (CNB) to confirm the benign nature; (iii) pressure symptoms or cosmetic problems caused by thyroid nodules; (iv) absence of any sonographic suspicious feature; and (v) follow-up for >6 months. The indication of thyroid RFA patients with benign thyroid nodules complaining of symptomatic or cosmetic problems. All patients had no contraindication for surgery, although they refused surgery over concerns of post-treatment scarring, complications, side effects, or thyroid functional changes.

### Pre-ablation Assessment

Clinical assessment before RFA included nodule-related symptom score, cosmetic score, and thyroid function. The nodule-related symptom score was obtained by patients filling out a questionnaire concerning five clinical symptoms: compression, cough, difficulty swallowing, voice change, and pain. For each positive symptom, we allocated one point, therefore the symptom scores ranged from 0 to 5. The cosmetic score was obtained using the following scale: 0, no visible or palpable mass; 1, not visible but palpable mass; 2, visible when swallowing only; 3, an easily visible mass. Patients with smaller neck circumference tended to complain about cosmetic problem earlier than those with thicker neck (13). Baseline laboratory data included serum thyroid-stimulating hormone (TSH), free thyroxine (fT4), and triiodothyronine (T3) levels.

Ultrasonography (US), US-guided FNAC, and US-guided CNB were performed by 2 radiologists (W.C.L. and K.L.C, each with more than 10 years of experience). Three orthogonal diameters of the thyroid nodule (the largest diameter and two other perpendicular ones) were measured by sonography. The volume of the thyroid nodule was calculated using the following equation: V = πabc/6 (V: volume; a: the largest diameter; b and c: the other two perpendicular diameters). Nodular composition was classified as solid, predominant solid, predominant cystic, or cyst (15, 16).

### Procedure

The ablation procedure was performed by 2 radiologists (W.C.L. and K.L.C). RFA was performed in an outpatient setting for all patients. Local anesthesia with a solution of 2% lidocaine hydrochloride buffered with sodium bicarbonate and epinephrine was injected at the puncture site and around the thyroid gland. In accordance with US examination guidelines, the electrode tip size was chosen based on tumor size and status of the surrounding critical structures. An internally cooled electrode (18 gauge, with 5, 7 mm or 1-cm active tip) with RF generator (VIVA, STARmed and M2004, RF Medical) were used. The trans-isthmic approach was used, passing through thyroid parenchyma with careful observation of the vessels along the approach route with aid of both gray-scale and color Doppler US. An electrode was inserted into the thyroid nodule at the deepest and most remote portion of the nodule. With application of the moving shot technique, the nodule was sequentially ablated. Ablation termination was determined when all conceptual ablation units of the nodule had changed to transient hyperechoic zones. Patients were continuously asked about the tolerability of the ablation during the procedure. Additional local anesthesia was injected if the patient complained of pain. After ablation, patients were routinely referred to the otolaryngology department to check for occurrence of vocal cord paralysis with flexible fiberoptic laryngoscopy.

### Follow-Up

US examinations and clinical symptoms were assessed in the same manner as those performed prior to the ablation procedure. Patients underwent US and clinical evaluations at 1-, 3-, and 6-month follow-up visits and at 6–12 months thereafter. Serum tests were performed at a 6-month follow up visit. Changes in largest nodule diameter and nodular volume were evaluated by US. The volume reduction rate was assessed by US imaging and was calculated by the following equation: volume reduction ratio (%) = initial volume (ml) − final volume (ml) × 100/initial volume. Symptoms and cosmetic scores were evaluated at each follow-up examination. Major and minor complications were assessed according to the standard terminology of the Society of Interventional Radiology (SIR) (14). Minor complications include SIR classification A–B; A: No therapy, no consequence; B: Nominal therapy, no consequence, includes overnight admission for observation only. Major complications include SIR classification C–F; C: Requires therapy, minor hospitalization (<48 h); D: Requires major therapy, prolonged hospitalization (>48 h); E: Permanent adverse sequelae; F: Death.

### Statistical Analysis

The statistical analysis was performed with the use of IBM SPSS, version 21 (Armonk, NY). Changes in largest nodule diameter, nodule volume, percentage volume reductions, and changes in symptom and cosmetic scores and serum tests during follow-up were compared with the use of Wilcoxon signed rank test. Statistical significance was accepted for *P* values < 0.05.

## Results

### Demographic Characteristics and Volume Reduction Ratio

**Figure 1:**
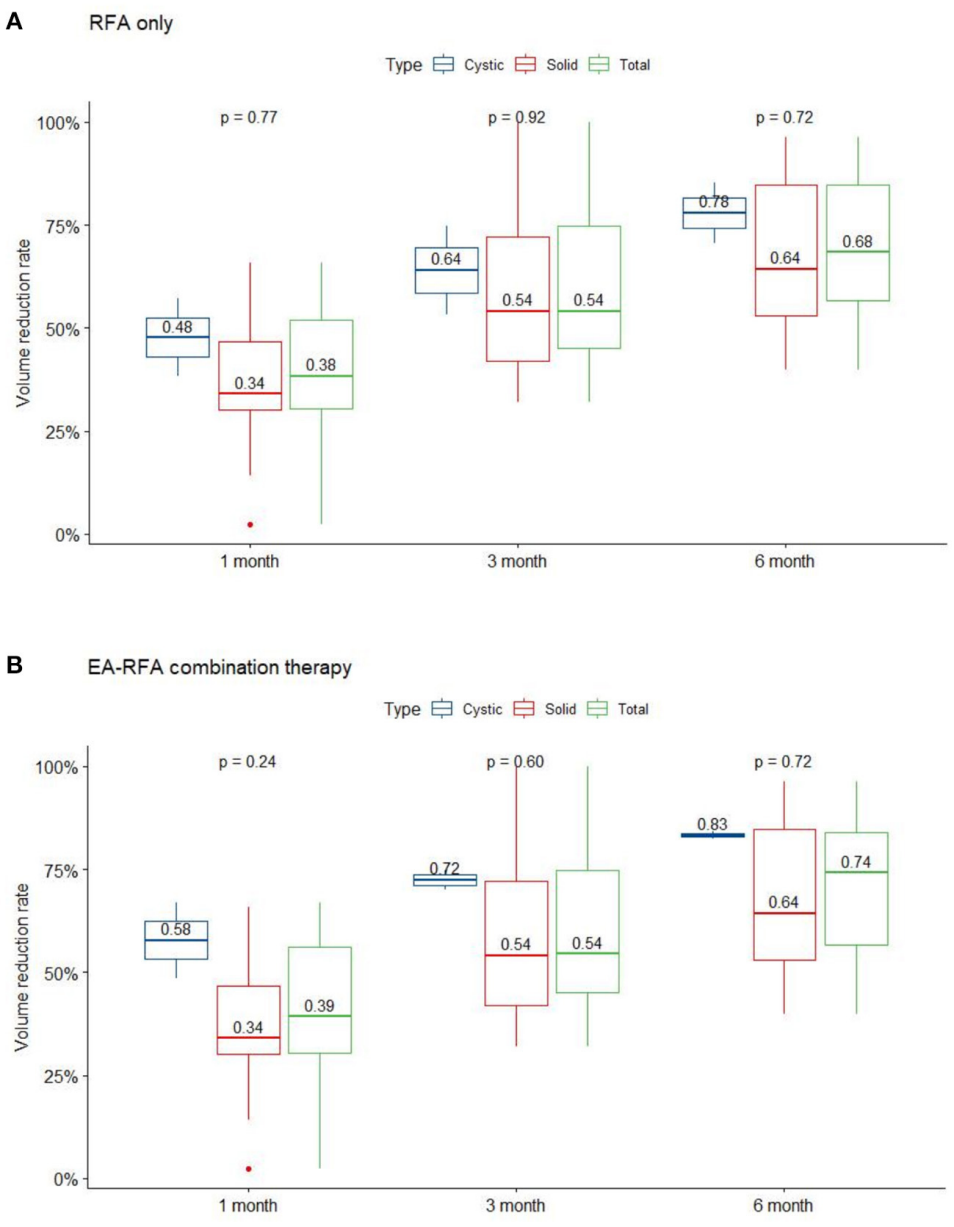
VRR changes of the three nodule composition groups of BTN at each follow-up. Changes in RFA only **(A)**, and EA-RFA combined therapy **(B)** of the different echogenicity groups of BTN at each follow-up. BTN, benign thyroid nodule; VRR, volume reduction ratio.

The demographic data of the 12 patients are presented in Table 1. All nodules were evaluated by sonography and defined as predominantly solid or predominantly cystic. Two patients with predominantly cystic lesions underwent ethanol sclerotherapy before the RFA procedure. Prior to ablation, the mean nodule volume was 15.11 ± 10.11 mL (range 0.84-29.88 mL). After ablation, mean nodule volume at 1, 3, and 6-month follow-ups were 8.94 ± 5.79, 6.31 ± 4.79, and 4.74 ± 3.75 mL, respectively. Mean volume reduction ratio (VRR) at 1, 3, and 6-month follow-ups were 40.2, 59.6, and 70.7%, respectively (Figure 1). At the last follow-up visit, the volume reduction rate was 74.31 ± 19.59%. There were 5 patients who underwent more than one session of thyroid RFA, one of whom was scheduled for staged therapy for right and left thyroid nodules. The mean interval between the first and second session of RFA was 320 ± 97.92 days.

**Table 1:** Profile of pediatric patients with benign thyroid nodules who underwent RFA.

**No**.	**Sex**	**Age (y)**	**Nodule characteristics**			**Number of ablations**	**Preablation symptom/cosmetic scores**	**6-month follow up symptom/cosmetic scores**	**Complication**
			**Diameter (cm)**	**Volume (mL)**	**Composition**				
1	F	13	5.7	28.65	Predominant solid	2	0/3	0/1	Transient vocal cord palsy
2	F	18	5.8	29.88	Solid	1	1/3	0/1	–
3	F	19	4.7	17.94	Predominant cyst	2	3/3	0/1	–
4	F	15	4.9	24.11	Solid	2	2/3	0/0	–
5	F	10	5.3	23.53	Predominant solid	3	2/3	0/1	–
6	F	12	1.5	1.73	Solid	1	0/3	0/1	–
7	F	15	3.6	9.8	Predominant cyst	1	0/3	0/0	–
8	F	18	2.3	2.31	Predominant solid	1	0/2	0/1	–
9	F	19	1.9	0.84	Solid	1	0/1	0/0	–
10	F	18	3.6	10.03	Solid	1	0/3	0/2	–
11	F	15	3.5	7.70	Solid	2	5/4	1/2	–
12	M	17	4.6	25.7	Solid	1	5/4	1/1	–
Mean		15.75	4.1	15.11			1.5/2.91	0.17/0.92	

### Symptom and Cosmetic Scores

The mean symptom and cosmetic scores were significantly reduced from baseline to 6-month follow-up: from 1.5 ± 1.93 to 0.17 ± 0.39 (*P* = 0.027), and from 2.91 ± 0.79 to 0.92 ± 0.67(*P* = 0.002), respectively. Serum free T4, T3, and TSH remained within normal limits before RFA and at 6-month follow-up.

### Complications and Management

**Figure 2:**
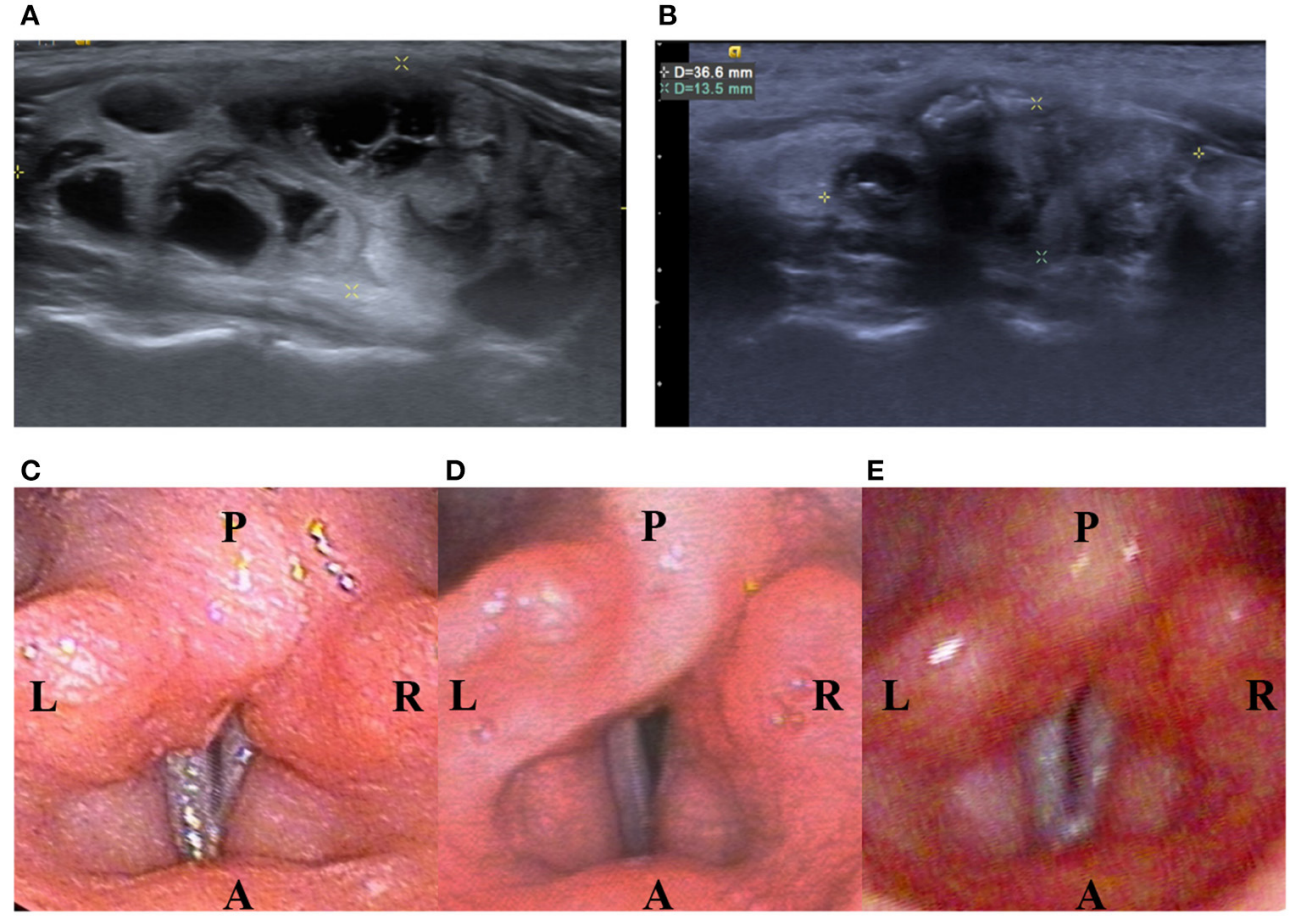
A 13-year-old female patient with a palpable nonfunctioning thyroid mass. **(A)** Before RF ablation, longitudinal sonographic views predominantly solid nodules in the right thyroid lobe. Initial nodule volume was 22.32 mL. **(B)** At 6 months after the ablation, a longitudinal sonographic view showed that the right thyroid nodule had shrunk. Nodule volume at 6-month follow-up was 4.65 mL, a volume reduction rate of 83%. **(C)** Before RF ablation, laryngoscope showed normal vocal cord. **(D)** On the same day after RF ablation, laryngoscope showed relative weakness of the right vocal cord but still with positive vibration. **(E)** On the 53rd day after RF ablation, laryngoscope showed normal vibration of bilateral vocal cord.

Several patients complained of neck pain and a heat sensation during the procedure, while mean intraoperative visual analog scale (VAS) was 4.2 ± 1.83 and mean postoperative VAS was 3.1 ± 2.21. There were no major complications such as nodule rupture or hypothyroidism noted during follow up. However, one patient was found to have voice weakness during RFA which did not recover after intravenous and perilesional methylprednisolone (40 mg) injection. Post-RFA laryngoscopy showed relatively weak right vocal cord vibration (Figure 2). The patient recovered spontaneously 53 days later without specific treatment.

## Discussion

### Summary and Solid Nodule VRR

**Figure 3:**
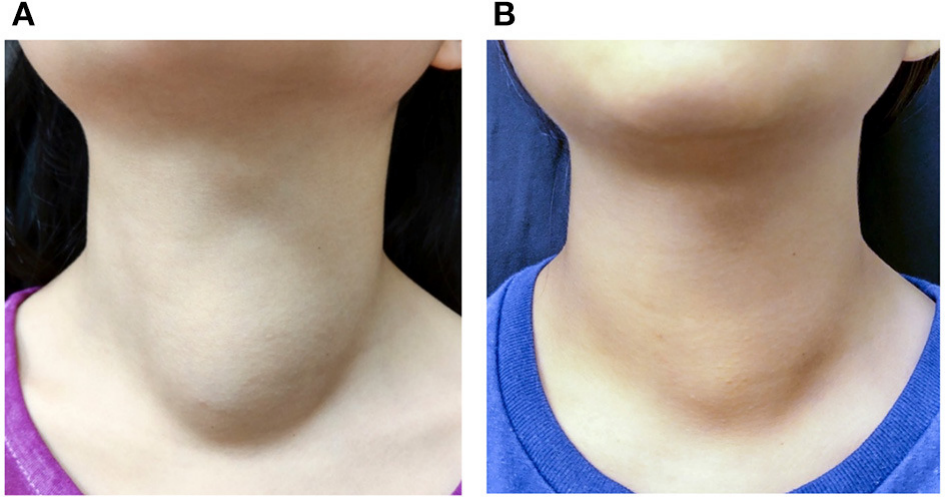
A 10-year-old female patient with a palpable nonfunctioning thyroid mass. **(A)** Before RF ablation, the cosmetic score was 3 and symptom score was 0. The initial nodule volume was 23.53 mL. **(B)** At 6 months after the ablation, the cosmetic score remained at 3 and symptom score was 0. The nodule volume was 15.99 mL.

Our study demonstrates the effectiveness of thyroid RFA in pediatric and adolescent patients. The VRR at the 6-month follow-up and final follow-up were 70.7 and 74.8%, respectively (Figure 3); whereas the only previous study of pediatric thyroid RFA reported the VRR at final follow-up was 92.1 ± 11.4% after 36.9 months mean follow-up (17). All of our patients had significant reductions in symptom and cosmetic scores. The currently limited evidence indicates that thyroid RFA could be an alternative therapy to manage symptomatic pediatric benign goiters, while further studies should be conducted in the future.

In this study, there were five patients who required multisession treatment due to residual/regrowth of the nodule. Several goiter characteristics may be associated with incomplete treatment and early regrowth of thyroid RFA, such as goiter baseline volume, vascularity, anatomical relationship to the critical structure, and ill-defined nodule goiters (18). In present study, no significant difference in baseline nodule volume between the single and multiple session treatment groups was noted (13.69 ± 11.70 vs. 18.32 ± 7.60, *p* = 0.205). Vascularity was not evaluated due to lack of data. The comparison of nodular components noted no significant difference between the predominantly solid and the cystic nodular components between the two groups. We identified one patient with multiple nodular goiters showing an ill-defined contour who required additional treatment.

Evidence of RFA for multiple nodular goiter is still limited and there long-term effect and safety is unknown. Multiple nodular goiter is related to PTEN and DICER gene mutation in pediatric patients which could further increase the risk of differentiated thyroid cancer such as papillary thyroid cancer and follicular thyroid cancer (19). Although the American College of Radiology Thyroid Imaging Reporting and Data System(TI-RADs) criteria is a recognized tool for thyroid nodules management in adults, its' inadequate for pediatric population since high percentage (22%) of cancer would have been missed due to not having FNAC (20). Also, techniques of tissue sampling such as aspiration and biopsy depends on operator's experience. Therefore, operation is indicated for multiple nodular goiter with compressive symptoms or occasionally cosmetic problems however the treatment strategy including the extent of thyroidectomy remains controversies (21–23). One of our patient had non-toxic multiple nodular goiter refused operation due to concerning about the complications such as laryngeal nerve injury and hypoparathyroidism. There was no suspicious feature of the multiple nodular goiter and benign nature was confirmed by twice core needle biopsy to dominant and non-dominant nodules respectively. Despite of the significant volume reduction effect of RFA, special caution should also be taken in non-operation management for pediatric multiple nodular goiter because of the high malignant potential. After RFA, the patient was followed up at outpatient department with regular sonography and repeated FNAs. We suggested that RFA should not be routinely performed in pediatric multiple nodular goiter without well planning.

Several factors associated with the RFA procedure could affect treatment effectiveness, such as operator technique and energy delivered to the goiter (24). A higher amount of heat is usually delivered to a solid nodule due to high tissue resistance. A previous study reported that a lower amount of energy delivered per milliliter of tissue (>0.219 kcal/mL) may be significantly associated with goiter regrowth (24). Although we herein delivered borderline higher energy/milliliter to the single session group compared to the multiple session group (0.789 ± 0.395 vs. 0.514 ± 0.131 kcal/mL, *p* = 0.089), the evidence is insufficient to indicate the energy effect in pediatric thyroid RFA (Table 2). Regarding the psychological aspect, the stress tolerance and mood status such as fear and anxiety during the RFA procedure could also alter the fluidity of the process and the final result. Thus, sufficient pre-procedural communication with the patient and family is important in order to balance treatment effectiveness and safety concerns.

**Table 2:** Comparison of potential factors affecting single and multiple session treatments.

	**Single session**	**Multiple session**	***P* value**
Baseline volume (mL)	13.69 ± 11.70	18.32 ± 7.60	0.205
Content (predominantly solid)	6	3	0.124
Energy delivery (kcal/mL)	0.789 ± 0.395	0.514 ± 0.131	0.089

### EA-RFA Combination Therapy

In patients with cystic and predominant cystic thyroid nodules, ethanol ablation (EA) is recommended as the first-line treatment due to its low cost, low technical difficulty, and high effectiveness (13, 25). The mean VRR of thyroid nodules with EA treatment was 91% (range: 83.1-96.9%) (26–28). However, according to a previous study, 26–33% of cases showed recurrence at the 1-month follow-up after EA therapy, with additional treatment required in 38.3% of patients (29). Regarding the factors related to poor treatment outcomes, the same study reported large initial nodular volume and presence of vascularity in solid components as independent factors. The solid component of the thyroid nodule was considered resistant to diffusivity, and prominent vascularity may facilitate drainage of ethanol, thereby limiting the effectiveness of EA treatment (29). RFA is suggested as the subsequent treatment for patients with recurrence or incomplete resolution due to its effective treatment of solid nodules. Two patients in our study with predominantly cystic thyroid nodules received EA-RFA combination therapy. The follow-up sonography after EA showed echogenic material with internal vascularity, indicating residual solid components. The mean VRR after EA was 13.2% and the mean VRR 6 months after EA-RFA combination therapy was 83.3%. Our study is consistent with previous studies which report the effectiveness of using EA-RFA combination therapy for predominantly cystic thyroid nodules (30–32).

### Safety and Complications

The only study focusing on pediatric thyroid RFA previously reported that the procedure is well-tolerated, with no major complications except for pain during the procedure (17). Meanwhile, an overall complication rate of 2.11% was reported in benign nodules in adults (13). Voice change is the most common complication after RFA, with incidence rates ranging from 0.5 to 4.7% (33). One of our patients experienced hoarseness during the procedure. The potential mechanisms of vocal cord paralysis are related to stretching of the nerve during the procedure, hemorrhage, or RFA-induced thermal injury rather than permanent nerve damage (14). Several methods have been recommended to avoid nerve injury, including hydrodissection between the nodule and the danger triangle, the maintenance of a minimal distance of more than 3 mm between the RF electrode and nerves, and undertreating the ablation units adjacent to the recurrent laryngeal nerve (34). Communication with patients intermittently during the procedure is important to assess voice condition and to treat the complication without delay (33).

As previously suggested, we used intravenous and local regional injection of steroids during the procedure to reduce post-operative neural edema and promote recovery of nerve function during voice change (35, 36). Although the patient's voice recovered completely within 2 months after the RFA procedure, the pharmacokinetic effect and relationship of steroids to nerve repair is unclear. Chung et al. report an immediate voice recovery by perineural cold dextrose solution injection during the procedure (37). A rapid decrease of the surrounding tissue temperature can prevent further nerve damage from the direct or indirect RFA-related heating effect and should be applied as early as possible in cases of voice change.

Thyroid RFA can be performed smoothly under local anesthesia in pediatric patients. There are two main stimuli during the procedure, including an initial skin puncture as well as the deeper pain associated with thermal tissue necrosis. To minimize these, we first buffered the lidocaine with sodium bicarbonate and epinephrine and used a small needle (24G) to reduce injection pain and prolong the duration of anesthesia (38, 39). Additionally, as sensory nerves are usually present at the thyroid capsule rather than inside the thyroid gland, appropriately performed perithyroidal lidocaine injection minimizes pain during the procedure (40). Moreover, as the anatomical location of the thyroid gland is shallow, the duration of the procedure is usually short. Of note, none of the patients in this study ended the procedure before the treatment was complete. Hence, thyroid RFA is a tolerable procedure in pediatric groups under local anesthesia.

### Limitations

Several limitations to the present study exist. First, it is limited by the small sample size. Second, the safety of thyroid RFA under local anesthesia in preschool children is currently a matter of debate. Although general anesthesia could prevent awareness during the procedure, voice condition cannot be monitored, which may increase the risk of hoarseness. Third, despite a benign cytology confirmed with at least 2 FNAs or core needle biopsies, the possibility of a false negative result or hidden malignancy cannot be overlooked due to the relatively higher malignancy rate in pediatric patients. There are currently no guidelines regarding follow-up intervals in pediatric patients. These risks warrant a cautious preoperational evaluation and long-term follow-up of pediatric patients. Last, due to the inherent limitations of retrospective single-center studies, further prospective multicenter and multinational studies are needed to confirm the results.

## Conclusion

In conclusion, RFA for non-functioning benign thyroid nodules is a safe and effective treatment for pediatric patients. For selective and cooperative patients, RFA could be an alternative treatment for thyroid goiters under local anesthesia.

## Data Availability Statement

The original contributions presented in the study are included in the article/supplementary material, further inquiries can be directed to the corresponding author/s.

## Ethics Statement

The studies involving human participants were reviewed and approved by Chang Gung Medical Foundation Institutional Review Board. Written informed consent to participate in this study was provided by the participants' legal guardian/next of kin.

## Author Contributions

K-LC, S-DL, P-LC, W-CC, C-KW, and Y-YS performed the measurements. W-CL was involved in planning and supervised the work. Y-SC and N-NK processed the experimental data, performed the analysis, and designed the figures. A-NL aided in interpreting the results and worked on the manuscript.

## Conflict of Interest

The authors declare that the research was conducted in the absence of any commercial or financial relationships that could be construed as a potential conflict of interest.

## Publisher's Note

All claims expressed in this article are solely those of the authors and do not necessarily represent those of their affiliated organizations, or those of the publisher, the editors and the reviewers. Any product that may be evaluated in this article, or claim that may be made by its manufacturer, is not guaranteed or endorsed by the publisher.
